# Factors associated with awareness of chronic kidney disease, and impact of awareness on renal prognosis

**DOI:** 10.1007/s10157-024-02605-4

**Published:** 2024-12-16

**Authors:** Akiko Hattori, Takahiro Imaizumi, Takuya Toda, Daisuke Sakurai, Nami Takai, Takahiro Miki, Michitaka Maekawa, Sawako Kato, Yuta Hagiwara, Yasuko Yoshida, Shoichi Maruyama

**Affiliations:** 1 Department of Nephrology, Nagoya University Graduate School of Medicine, 65 Tsurumai-Cho, Showa-Ku, Nagoya, 466-8550 Japan; 2 Department of Advanced Medicine, Nagoya University Hospital, Nagoya, Japan; 3 PREVENT Inc., Nagoya, Japan; 4 Department of Nursing, Nagoya University Hospital, Nagoya, Japan; 5 Hidamari Kokoro Clinic, Nagoya, Japan; 6 Center for Research, Education, and Development of Healthcare Life Design, Tokai National Higher Education and Research System, Nagoya, Japan; 7 Department of Innovative Research Center for Preventive Medical Engineering, Nagoya University Graduate School of Medicine, Nagoya, Japan

**Keywords:** Awareness, CKD, eGFR slope, Nutritional guidance, Urine tests

## Abstract

**Background:**

Chronic kidney disease (CKD) awareness could help prevent disease progression through modifiable risk factors. However, few patients with CKD are aware of their disease. We aimed to investigate the factors associated with CKD awareness and impact of CKD awareness on renal prognosis.

**Methods:**

We investigated the proportion of participants with CKD who answered ‘aware of CKD’ in the questionnaire among those undergoing health check-ups from 2013 to 2022. Participants included working-age employees and their dependents covered by health insurance associations for large and medium-sized companies. The outcome was defined as the change from ‘unaware’ to ‘aware’ of CKD; multivariable logistic regression analysis assessed the association of urine tests or nutritional guidance with CKD awareness. A control group was randomly selected from the unaware group and matched for age, sex, estimated glomerular filtration rate (eGFR), urinary protein categories, and follow-up period. Changes in eGFR slopes before and after awareness were compared using linear mixed-effects models.

**Results:**

Of the 13,489 participants, 2.8% were aware of CKD at baseline; of the 1,614 with CKD-related disease codes, only 19.6% were aware. The odds ratios of urine tests or nutritional guidance in relation to awareness occurrence were 1.98 (1.29–3.05) and 3.01 (1.38–6.53), respectively. The difference in the eGFR slope improvement from before to after CKD awareness was + 0.92 mL/min/1.73 m^2^ per year (0.18–1.67; *P* = 0.015) in the aware group.

**Conclusion:**

Our findings suggest that urine tests and nutritional guidance may promote CKD awareness, which may help slow its progression.

**Supplementary Information:**

The online version contains supplementary material available at 10.1007/s10157-024-02605-4.

## Introduction

The global prevalence of chronic kidney disease (CKD) is 13.4% and expected to rise with the increasing prevalence of diabetes mellitus (DM), hypertension (HT), and aging population [1, 2]. Globally, CKD ranked 7th among mortality risk factors in 2019, highlighting the importance for combatting CKD [3]. The increasing prevalence of CKD is a serious problem not only in low- and middle-income countries [4, 5] but also in high-income countries, including Japan [6]. In Japan, which has a large number of patients on dialysis, the number of patients with non-dialysis-dependent CKD is on the rise [7, 8]. Recent reports have indicated that medications such as sodium-glucose cotransporter 2 inhibitors (SGLT2-i) slow CKD progression [9, 10] in combination with multidisciplinary care [11]. Thus, in this era, CKD progression and its complications are preventable through proactive medical interventions.

Despite the increasing potential for medical intervention, the recognition of CKD in the general population remains low [12, 13]; in various regions, only 7–20% of patients with CKD were aware of their CKD status [3, 14–16]. In a study of specific health check-ups for participants aged 40–74 years using the National Database of Health Insurance Claims and Specific Health Checkups of Japan (NDB) Open Data Japan database, the percentage of CKD awareness was only 0.57% [17]. Lifestyle factors, including smoking, diet, and exercise, are common risk factors for CKD onset and progression [18–20]. Therefore, lifestyle modifications may reduce the risk of CKD development and progression. However, CKD is asymptomatic, which makes an early-stage CKD diagnosis unlikely [15] and further limits the opportunities for early intervention. Awareness is higher among those with advanced-stage CKD [15, 16], complicating efforts to demonstrate the efficacy of CKD awareness in slowing CKD progression.

Factors that may promote CKD awareness can be categorized into patient, medical provider, and social system factors. Among these, the identification of medical provider factors that could promote patient awareness of early-stage CKD may facilitate appropriate preventive care. Among the possible candidate medical practices for awareness, we focused on factors commonly used as quality indicators for CKD care [21, 22], particularly urine tests and nutritional guidance, which are accessible through insurance claims data.

We aimed to investigate whether the utilization of urine tests and nutritional guidance was associated with occurrence of CKD awareness. Furthermore, we examined the impact of CKD awareness on renal prognosis.

## Materials and methods

### Data source and study population

**Fig. 1:**
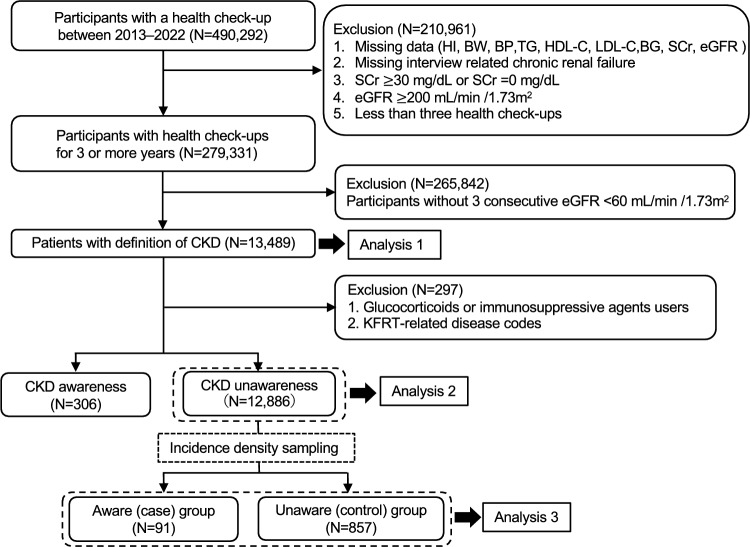
Flowchart of the study design and participant selection. *CKD* chronic kidney disease, *HI* height, *BW* body weight, *BP* blood pressure, *TG* triglyceride, *HDL-C* high-density lipoprotein cholesterol, *LDL-C* low-density lipoprotein cholesterol, *BG* blood glucose, *SCr* serum creatinine, *eGFR* estimated glomerular filtration rate, *KFRT* kidney failure with replacement therapy

From the PREVENT Inc. (Nagoya, Japan) database, we obtained annual health check-up data and reviewed the medical claims records of workers and their dependents. These data were collected from approximately 40 health insurance associations of large and medium-sized general companies in Japan. The database comprised records of adults (age ≥ 18 years) who underwent a health check-up between 2013 and 2022. Health check-up data included two types of health check-ups: specific and workplace health check-ups. Medical claims records included inpatient, outpatient, and dispensing claims records. We excluded participants with missing data on essential covariates; a questionnaire on chronic kidney failure; outliers in serum creatinine (SCr) and estimated glomerular filtration rate (eGFR) values; and fewer than three health check-ups (Fig. 1). This study followed the principles of the Declaration of Helsinki; the study protocol was approved by the Ethics Review Committee of Nagoya University Hospital (approval no. 2020-0142), which waived the requirement for written informed consent owing to the anonymized data use.

### Definition and study design

In this study, CKD was defined as three consecutive eGFR values < 60 mL/min/1.73 m^2^, which was calculated using the following formula developed for the Japanese population [23, 24]:

$$\text{eGFR }(\text{mL}/\text{min}/1.73 {\text{m}}^{2}) =194 \times \text{ SC}{\text{r}}^{-1.094}(\text{mg}/\text{dL}) \times {\text{age}}^{-0.287} (\text{years}) \,(\times 0.739\text{ for females})$$

The baseline was defined as the first year of three consecutive years in which the eGFR < 60 mL/min/1.73 m^2^. We defined awareness of CKD as an affirmative response (YES) to the health check-up questionnaire item: ‘Have you ever been diagnosed with chronic kidney failure by a doctor or received treatment (dialysis)?’ Furthermore, we defined occurrence of awareness as the conversion of awareness status from unawareness at baseline to awareness in the following year.

We separately performed three analyses: Analysis 1, which involved a cross-sectional descriptive analysis at baseline; Analysis 2, which pertained to the identification of factors associated with the occurrence of awareness; and Analysis 3, which was a longitudinal analysis of differences in the improvement of the eGFR slope between the aware and unaware groups. Among the identified participants with CKD, those who used glucocorticoids or immunosuppressive agents within a year before baseline or had kidney failure with replacement therapy (KFRT)-related codes were excluded in Analyses 2 and 3. In Analysis 1, we described the prevalence of CKD awareness and CKD-related diseases codes at baseline; the baseline characteristics were compared between the CKD-aware and unaware groups. In Analysis 2, we examined clinical characteristics and the impact of urine tests or nutritional guidance on CKD awareness in the following year within the baseline CKD-unaware group. The baseline characteristics were compared by whether urine tests or nutritional guidance were provided in the following year of the baseline. The practices were identified using the medical practice codes of the Japanese health insurance system (Supplementary Table S1). In Analysis 3, to compare changes in the eGFR slope between the participants with and without CKD awareness, using incidence density sampling, we matched up to 10 controls from the study cohort to each case by age (± 3 years), sex, eGFR (± 5 mL/min/1.73 m^2^), urinary protein categories (≥ 1 + , ± / −, or missing), and follow-up period (Supplementary Fig. S1). The index year was defined as the year in which the participants in the case group became aware of their CKD. In the control group, the corresponding year from the baseline was considered the index year. After incidence density sampling, we excluded participants with only one health check-up after the index year or fewer than five controls per case, along with their corresponding controls. For the case–control comparison of differences in the annual changes in the eGFR slope before and after the index year, we used a linear mixed-effects model with individuals as a random effect [25]. The change in participants' motivation for lifestyle modification before and after the index year was evaluated by the percentage of responses to the question, ‘Do you want to improve your lifestyle habits, such as exercise and diet?’.

### Covariates

In Analysis 2, to examine the association of baseline data and subsequent prescription information with the occurrence of awareness in the following year, we defined DM, HT, and dyslipidemia as follows: DM was defined as a fasting blood glucose ≥ 126 mg/dL on baseline data or the prescription of medications for DM during the year leading up to the next health check-up; HT was defined as a blood pressure ≥ 140/90 mmHg or the prescription for HT; and dyslipidemia was defined as an LDL cholesterol ≥ 140 mg/dL, HDL cholesterol < 40 mg/dL, or triglycerides ≥ 150 mg/dL or the prescription for dyslipidemia. In Analysis 3, health check-up data in the index year and the prescription information in the preceding year were used to define DM and HT. Prescription data on SGLT2-i, renin-angiotensin system inhibitors (RAS-i), glucocorticoids, and immunosuppressive agents use were identified using drug codes from the medical claims, in the year before each health check-up (Supplementary Table S2). CKD-related disease codes were defined as those assigned by doctors, reflecting their recognition of CKD. We used CKD or KFRT-related codes from the International Classification of Diseases, Tenth Revision, 2013, based on previous studies and expert reports (Supplementary Table S3) [26, 27].

### Statistical analysis

**Fig. 2:**
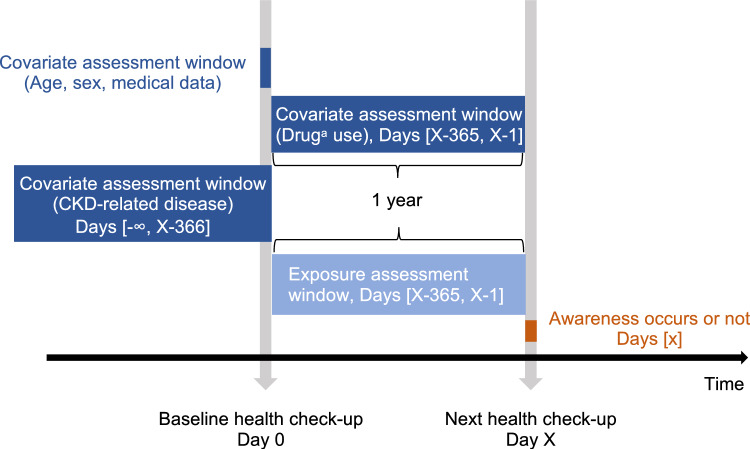
Study design in Analysis 2. ^a^Drugs included medications for hypertension, diabetes mellitus, or dyslipidemia. *CKD* chronic kidney disease, *eGFR* estimated glomerular filtration rate

Baseline characteristics in Analyses 1 and 2, and the participants' characteristics in the index year in Analysis 3 were described as the frequency (%) or mean ± standard deviation. In Analysis 2, multivariable logistic regression was used to analyze the association of urine testing or nutritional guidance in the preceding year with occurrence of CKD awareness in the newly aware and unaware groups. The objective variable was occurrence of CKD awareness, and the explanatory variables were urine tests or nutritional guidance. We adjusted for age, sex, eGFR, previous CKD-related disease codes, DM, HT, dyslipidemia, body mass index (BMI), urinary protein, and smoking status (Fig. 2). In the sensitivity analysis, new CKD-related disease codes diagnosed contemporaneously with urine tests or nutritional guidance were added as covariates instead of previous codes. There was a large number of missing data on urinary protein, thereby we classified the data into ≥ 1 + , ± or −, and missing for analysis. Additionally, the frequency of the practices was categorized to determine their association with CKD awareness. Urine tests were stratified as 0, 1–3, 4–6, and ≥ 7, and nutritional guidance as 0, 1, and ≥ 2 times per year because few participants received it ≥ 2 times. In subgroup analysis, we examined whether SCr was measured within a year before baseline, as a proxy for outpatient visits regardless of CKD care. In Analysis 3, we used linear mixed-effects models with an unstructured variance–covariance matrix, random intercept, and random slope to calculate the eGFR slope [28]. We adjusted for time-updated age, sex, DM, HT, and time-updated BMI with the interaction between the respective covariates and time. The eGFR in the index year was added to the model without any interaction with time. We conducted sensitivity analysis to consider the impact of medical interventions. Specifically, we added the time-updated use of SGLT2-i, RAS-i, glucocorticoids, and immunosuppressive agents and CKD-related disease codes newly added during the preceding year leading up to the index year, along with their interaction with time. Changes in the participants' motivation were tested using the Jonckheere-Terpstra trend test. All analyses were performed using Stata/MP version 17.0 (StataCorp, USA). *P* < 0.05 was considered statistically significant.

## Results

Among the 490,292 participants, 13,489 were identified with CKD. After excluding the use of glucocorticoids or immunosuppressive agents and those with KFRT-related disease codes, 13,192 participants were identified for Analyses 2 and 3 (Fig. 1). Of these, 12,886 were CKD-unaware at baseline, 111 were in the newly aware group who became aware of the next health check-up; 12,775 were in the unaware group who remained unaware (Analysis 2). Of the 2699 identified using density sampling, 1,608 with one health check-up after the index year, and further 143 with fewer than five controls per case, were excluded. Finally, 948 (case, N = 91; control, N = 857) were identified (Analysis 3).

### Analysis 1: prevalence of CKD awareness

Of the 13,489, 372 (2.8%) were aware of their CKD. During the following year, 2397 (17.8%) underwent a urine test and 226 (1.7%) received nutritional guidance. The aware group had lower eGFR and more DM and HT at baseline. The prevalence of CKD-related disease codes was 1614 (12.0%), of which only 316 (19.6%) were CKD-aware (Supplementary Table S4 and Supplementary Fig. S2).

### Analysis 2: association of urine tests or nutritional guidance with the occurrence of CKD awareness

**Fig. 3:**
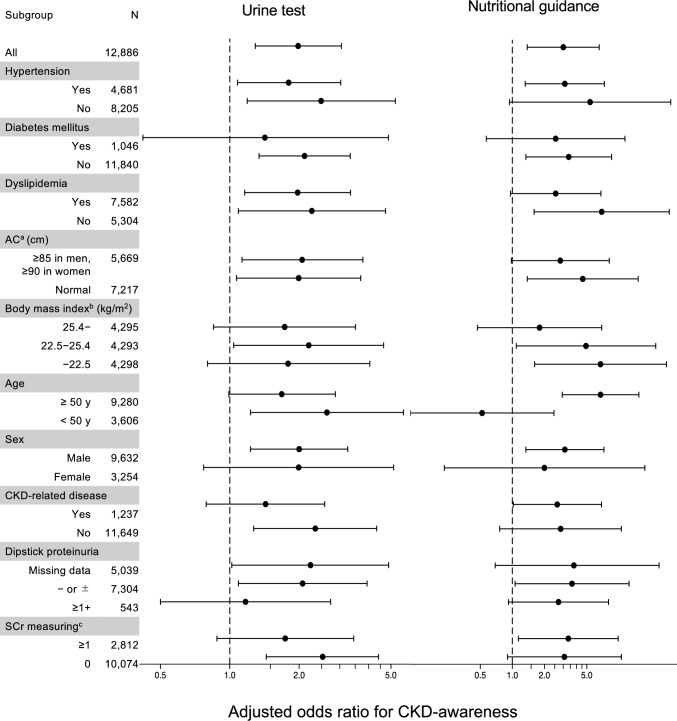
Multivariable logistic regression and subgroup analysis. Forest plots showing the adjusted odds ratio (95% CI) of the association of the urine tests or nutritional guidance with the occurrence of CKD awareness. **a** AC, classified according to Japanese Metabolic Syndrome Criteria. **b** Based on the body mass index, participants were subcategorized into three groups using the tertile method. **c** SCr measuring, frequency of serum creatinine measurement in a past year before baseline. *CI* confidence interval, *AC* abdominal circumference, *CKD* chronic kidney disease, *SCr* serum creatinine

The mean eGFR in participants who were CKD-unaware at baseline was 54.2 mL/min/1.73 m^2^; 97.5% of the participants had 40 ≤ eGFR < 60 mL/min/1.73 m^2^. In the following year, 2270 (17.6%) received urine tests; 168 (1.3%) received nutritional guidance. Those who underwent urine testing or nutritional guidance had higher comorbidities, including DM, dyslipidemia, HT, or CKD-related disease codes (Table 1). In addition, participants who received nutritional guidance had poorer kidney function (48.9 ± 11.9 vs. 54.3 ± 5.38 mL/min/1.73 m^2^). The adjusted odds ratios (OR) [95% confidence interval (CI)] of urine tests and nutritional guidance associated with CKD awareness were 1.98 [1.29–3.05, *P* = 0.002] and 3.01 [1.38–6.53, *P* = 0.005], respectively (Table 2). Sensitivity analysis using new CKD-related disease codes as covariates showed similar results. Regarding the relationship between categorical urine tests and CKD awareness, the OR increased in a dose–response manner, except for participants with sessions ≥ 7 times per year. With nutritional guidance, the adjusted OR [95% CI] was 2.55 [0.79–8.31,* P* = 0.119] and 3.38 [1.27–9.04, *P* = 0.015] for 1 or ≥ 2 times per year, respectively (Supplementary Tables S5 and S6). In the subgroup analysis, the OR was attenuated in the groups with DM, HT, dyslipidemia, and obesity. In addition, association with awareness and urine tests was weaker in the group with SCr measurement (OR: 1.74 (95%CI 0.88–3.45; *P* = 0.113) vs. OR: 2.53 (95%CI 1.44–4.42; *P* = 0.001); Fig. 3).

**Table 1:** Baseline characteristics of participants with CKD who were unaware at baseline

Variables	Total	Urine test (times/year)		Nutritional guidance (times/year)	
		≥ 1	0	≥ 1	0
	N = 12,886	N = 2,270	N = 10,616	N = 168	N = 12,718
Male, n (%)	9,632 (74.7)	1,737 (76.5)	7,895 (74.4)	139 (82.7)	9,493 (74.6)
Age, years (mean ± SD)	53.5 ± 6.8	54.0 ± 6.8	53.3 ± 6.7	53.1 ± 7.7	53.5 ± 6.7
Body mass index, kg/m^2^ (mean ± SD)	24.2 ± 3.6	24.8 ± 3.8	24.1 ± 3.5	26.6 ± 4.7	24.2 ± 3.5
Systolic blood pressure, mmHg (mean ± SD)	124 ± 17	126 ± 17	123 ± 17	131 ± 19	123 ± 17
Diastolic blood pressure, mmHg (mean ± SD)	78 ± 12	79 ± 12	78 ± 12	82 ± 13	78 ± 12
Triglycerides, mg/dL (mean ± SD)	124.9 ± 89.9	133.4 ± 102.8	123.1 ± 86.8	170.9 ± 123.4	124.3 ± 89.2
HDL-C, mg/dL (mean ± SD)	61.1 ± 17.3	59.7 ± 16.7	61.4 ± 17.4	54.3 ± 13.6	61.2 ± 17.3
LDL-C, mg/dL (mean ± SD)	128.6 ± 30.8	126.4 ± 31.9	129.0 ± 30.5	131.5 ± 34.1	128.5 ± 30.7
Fasting blood glucose, mg/dL (mean ± SD)	99.8 ± 17.4	105.2 ± 23.8	98.6 ± 15.5	117.6 ± 34.8	99.6 ± 17.0
Serum creatinine, mg/dL (mean ± SD)	1.08 ± 0.28	1.12 ± 0.29	1.07 ± 0.28	1.45 ± 1.30	1.08 ± 0.24
eGFR, mL/min/1.73 m^2^ (mean ± SD)	54.2 ± 5.55	52.8 ± 6.61	54.5 ± 5.25	48.9 ± 11.9	54.3 ± 5.38
eGFR category, mL/min/1.73 m^2^, n (%)					
< 30	93 (0.7)	28 (1.2)	65 (0.6)	13 (7.7)	80 (0.6)
30–39	226 (1.8)	75 (3.3)	151 (1.4)	10 (6.0)	216 (1.7)
40–49	1,671 (13.0)	440 (19.4)	1,231 (11.6)	43 (25.6)	1,628 (12.8)
50–59	10,896 (84.6)	1,727 (76.1)	9,169 (86.4)	102 (60.7)	10,794 (84.9)
Dipstick proteinuria category, n (%)					
Missing data	5,039 (39.1)	810 (35.7)	4,229 (39.8)	46 (27.4)	4,993 (39.3)
Negative (−) or trace ( ±)	7,304 (56.7)	1,299 (57.2)	6,005 (56.6)	89 (53.0)	7,215 (56.7)
Positive (≥ 1 +)	543 (4.2)	161 (7.1)	382 (3.6)	33 (19.6)	510 (4.0)
Current smoker, n (%)	2,228 (17.3)	386 (17.0)	1,842 (17.4)	41 (24.4)	2,187 (17.2)
Diabetes mellitus, n (%)	1,046 (8.1)	458 (20.2)	588 (5.5)	67 (39.9)	979 (7.7)
Dyslipidemia, n (%)	7,582 (58.8)	1,544 (68.0)	6,038 (56.9)	138 (82.1)	7,444 (58.5)
Hypertension, n (%)	4,681 (36.3)	1,231 (54.2)	3,450 (32.5)	115 (68.5)	4,566 (35.9)
CKD-related disease codes, n (%)	1,239 (9.6)	464 (20.4)	775 (7.3)	58 (34.5)	1,181 (9.3)
SCr measurement, times /year, n (%)					
0	10,074 (78.2)	1,281 (56.4)	8,793 (82.8)	86 (51.2)	9,988 (78.5)
1–3	2,256 (17.5)	755 (33.3)	1,501 (14.1)	47 (28.0)	2,209 (17.4)
4–6	395 (3.1)	159 (7.0)	236 (2.2)	19 (11.3)	376 (3.0)
≥ 7	161 (1.2)	75 (3.3)	86 (0.8)	16 (9.5)	145 (1.1)

Data are expressed as the frequency (%) or mean ± standard deviation (SD)

*HDL-C* high-density lipoprotein cholesterol, *LDL-C* low-density lipoprotein cholesterol, *eGFR* estimated glomerular filtration rate, *CKD* chronic kidney disease, *SCr* serum creatinine

**Table 2:** Association of the urine tests or nutritional guidance with the occurrence of CKD awareness in participants with unawareness at baseline

Clinical care	Unadjusted		Adjusted^a^		Sensitivity analysis^b^	
	OR (95% CI)	*P*-value	OR (95% CI)	*P*-value	OR (95% CI)	*P*-value
Urine test	3.23 (2.21–4.74)	< 0.001	1.98 (1.29–3.05)	0.002	2.10 (1.36–3.26)	0.001
Nutritional guidance	11.8 (6.61–21.2)	< 0.001	3.01 (1.38–6.53)	0.005	2.60 (1.15–5.85)	0.021

Odds ratios (OR) of the association of at least one urine test or nutritional guidance session with awareness

^a^We adjusted for age, sex, estimated glomerular filtration rate (eGFR), previous CKD-related disease codes, diabetes mellitus (DM), hypertension (HT), dyslipidemia, body mass index (BMI), urinary protein, and smoking in the main analysis

^b^In the sensitivity analysis, we adjusted for age, sex, eGFR, new CKD-related disease codes (diagnosed contemporaneously with a urine test or nutritional guidance), DM, HT, dyslipidemia, BMI, urinary protein, and smoking. *CKD* chronic kidney disease, *CI* confidence interval

### Analysis 3: difference in the change of the eGFR slopes before and after CKD awareness

**Fig. 4:**
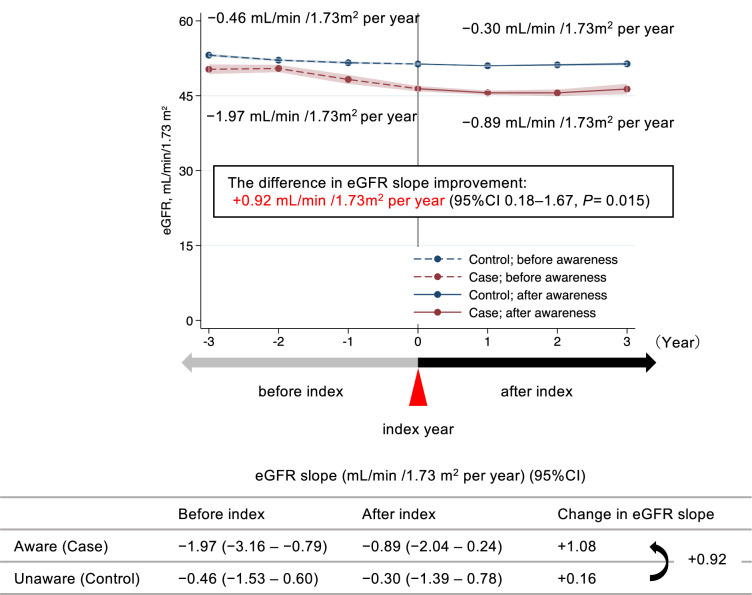
Difference in the change of the eGFR slopes between case and control groups before and after the occurrence of awareness. The index year was defined as the year in which the patients were aware of chronic kidney disease (CKD). We adjusted for time-updated age, sex, diabetes mellitus, hypertension, and time-updated body mass index, with interactions between the respective covariates and time. The eGFR in the index year was added without any interaction with time. The blue line represents the unaware (control) group and the red line represents the aware (case) group. The dotted and solid lines show the eGFR slope before and after the index year, respectively. *eGFR* estimated glomerular filtration rate, *CI* confidence interval

**Fig. 5:**
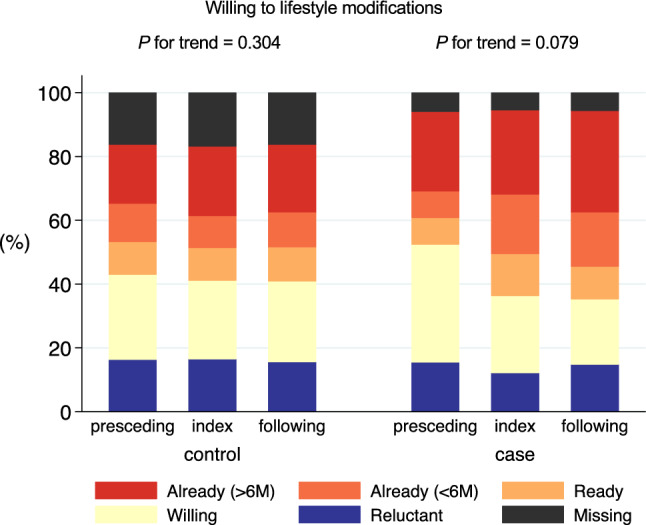
Change in participant’s attitudes before and after the index year. Percentage of responses to the health check-up question,’Do you want to improve your lifestyle habits, such as exercise and diet?’ The results were tested using the Jonckheere-Terpstra trend test

The participant characteristics at the index year showed that the case group had low eGFR (46.0 ± 9.20 vs. 51.3 ± 6.15 mL/min/1.73 m^2^; *P* < 0.001), and used more RAS-i (Table 3). The eGFR slope before and after the index year in the case group was − 1.97 and − 0.89 mL/min/1.73 m^2^ per year, respectively, with a change of + 1.08 mL/min/1.73 m^2^ per year. In the control group, the eGFR slope before and after the index year was − 0.46 and − 0.30 mL/min/1.73 m^2^ per year, respectively, with a change of + 0.16 mL/min/1.73 m^2^ per year. Thus, the difference in the eGFR slope improvement from before to after CKD awareness was + 0.92 mL/min/1.73 m^2^ per year (95% CI 0.18–1.67; *P* = 0.015) in the case group (Fig. 4). In the sensitivity analysis, the difference in the eGFR slope improvement in the case group was + 0.93 mL/min/1.73 m^2^ per year (95% CI 0.18–1.68; *P* = 0.014; Supplementary Fig. S3). The results of the questionnaire analysis showed that 36.4% of the control group had already practiced lifestyle modification in the pre-index year, with similar levels of 38.3% and 38.3% in the index and following years, respectively. In contrast, the case group changed from 35.4% in the pre-index year to 47.7% and 51.8% in the index and subsequent years, respectively (*P* = 0.079; Fig. 5).

**Table 3:** Participants’ characteristics at index year

Variables	Total	Aware	Unaware
	N = 948	N = 91	N = 857
Male, n (%)	727 (76.7)	70 (76.9)	657 (76.7)
Age, years (mean ± SD)	55.7 ± 5.8	54.0 ± 5.9	55.9 ± 5.7
Body mass index, kg/m^2^ (mean ± SD)	24.4 ± 3.7	24.3 ± 4.1	24.4 ± 3.7
Systolic blood pressure, mmHg (mean ± SD)	125 ± 17	123 ± 15	125 ± 17
Diastolic blood pressure, mmHg (mean ± SD)	79 ± 12	78 ± 10	79 ± 12
Triglycerides, mg/dL (mean ± SD)	130.2 ± 98.8	131.9 ± 83.0	130.0 ± 100.4
HDL-C, mg/dL (mean ± SD)	60.4 ± 17.1	60.4 ± 18.1	60.5 ± 17.0
LDL-C, mg/dL (mean ± SD)	124.9 ± 30.4	120.5 ± 31.0	125.4 ± 30.3
Fasting blood glucose, mg/dL (mean ± SD)	102.0 ± 19.7	100.9 ± 18.9	102.1 ± 19.8
Serum creatinine, mg/dL (mean ± SD)	1.15 ± 0.35	1.36 ± 0.81	1.13 ± 0.24
eGFR, mL/min/1.73 m^2^ (mean ± SD)	50.8 ± 6.68	46.0 ± 9.20	51.3 ± 6.15
Dipstick proteinuria category, n (%)			
Missing data	424 (44.7)	39 (42.9)	385 (44.9)
Negative (−) or trace ( ±)	464 (48.9)	45 (49.5)	419 (48.9)
Positive (≥ 1 +)	60 (6.3)	7 (7.7)	53 (6.2)
Current smoker, n (%)	139 (14.7)	11 (12.2)	128 (14.9)
Diabetes mellitus, n (%)	109 (11.5)	11 (12.1)	98 (11.4)
SGLT2 inhibitors, n (%)	27 (2.8)	3 (3.3)	24 (2.8)
Hypertension, n (%)	420 (44.3)	50 (54.9)	370 (43.2)
RAS inhibitors, n (%)	215 (22.7)	40 (44.0)	175 (20.4)
Glucocorticoids or immunosuppressive agents, n (%)	19 (2.0)	5 (5.5)	14 (1.6)
CKD-related disease codes, n (%)	30 (3.2)	10 (11.0)	20 (2.3)

Data are expressed as the frequency (%) or mean ± standard deviation (SD). *HDL-C* high-density lipoprotein cholesterol, *LDL-C* low-density lipoprotein cholesterol, *eGFR* estimated glomerular filtration rate, *SGLT2* sodium-glucose cotransporter 2, *RAS* renin-angiotensin system, *CKD* chronic kidney disease

## Discussion

This study indicated that only 2.8% of participants had CKD awareness, despite the stringent criteria for CKD defined by three consecutive eGFR values < 60 mL/min/1.73 m^2^. Notably, 98.6% of participants with CKD Stage 3 were CKD-unaware, indicating the importance of a more proactive approach for early-stage CKD. Urine tests or nutritional guidance in the preceding year was associated with the occurrence of CKD awareness. More participants became CKD-aware when the practices were conducted multiple times. This novel study demonstrates the importance of proactive medical practices by medical providers for promoting CKD awareness and shows that CKD awareness was associated with slower eGFR decline. To our knowledge, this is the first study to identify a significant association between CKD awareness and slowing kidney function decline. Subgroup analysis showed that the association between CKD awareness and the medical practices was weakened in individuals with comorbidities, including DM, HT, dyslipidemia, and obesity. In addition, for those who visit the hospital regularly, simply performing urine tests was less effective in developing CKD awareness. However, these results do not mean that the performance of these tests was insufficient and, instead, suggest the importance of individualized treatment approaches based on patients’ backgrounds rather than a one-size-fits-all approach.

The magnitude of these practices is substantial. In this study, the numbers needed to treat for promoting awareness through urine tests and nutritional guidance were 71.4 and 13.3, respectively. Urine tests provide useful information for diagnosing CKD and predicting prognosis [29, 30]. Those who received nutritional guidance were less likely to drop out of diabetes visits [31]. Thus, proactive interventions promote disease-awareness and motivate patients to continue seeing their doctors. Similar mechanisms may exist in patients with CKD. However, this study found that these practices were underutilized in patients with CKD. Further research is required to determine how to expand the use of them.

The benefits of CKD awareness include lifestyle modification and avoidance of dehydration or nephrotoxic substances, which can slow CKD progression [15, 16, 32]. However, a previous study found that patients with CKD awareness had higher rates of KFRT and mortality [16], possibly due to a reversal of cause and effect, where poor prognosis led to increased awareness. To avoid such causal reversal, we evaluated the impact of awareness by comparing eGFR slope changes before and after awareness in individuals with earlier-stage CKD than in previous studies, after adjusting for relevant clinical factors. Nevertheless, the rate of eGFR decline was greater in the case group than in the control group both before and after the index. This may be because those with the rapid eGFR decline is more likely to be aware. Moreover, unmeasured confounders might still have affected the eGFR decline, even after adjusting for as many covariates as possible at the index. The post-awareness eGFR trajectory was not observed for a long enough period of time in the present data. Further long-term observation is needed to clarify whether the eGFR slope could improve more in the aware group than in the unaware group.

This study showed an improvement in the eGFR slope by + 1.08 mL/min/1.73 m^2^ per year after CKD awareness, highlighting its importance in early-stage CKD. Although previous studies showed that multidisciplinary care was associated with CKD improvement (eGFR > 4 mL/min/1.73 m^2^) [33–35], most patients in these studies had stage 4 or 5 CKD. In contrast, the mean eGFR of the aware group in this study was 46.0 mL/min/1.73 m^2^, making the improvement more modest than in previous studies of multidisciplinary care. Nonetheless, even a 0.75 mL/min/1.73 m^2^ per year improvement in the eGFR slope can significantly reduce the risk of KFRT [28]. Therefore, the contribution of CKD awareness in improving the eGFR slope in this study was large enough to indicate a potential benefit in renal prognosis.

Our study has some limitations. First, selection bias might exist because patients with comorbidities or advanced CKD were less likely to undergo health check-ups, resulting in a sample biased toward early-stage CKD and potentially underestimating awareness. However, earlier-stage intervention is desirable to prevent CKD progression, making this focus appropriate. Second, we were unable to determine whether participants visited a nephrologist or primary care physician, which may affect the impact of urine tests or nutritional guidance. Nonetheless, our results suggest that these practices contributed significantly to promoting awareness. Third, participants with stage 1 or 2 CKD were excluded because we did not use a urinary protein-based CKD definition. Approximately 10% of patients in the Japan CKD Database (J-CKD-DB) [36] had these stages; not including these patients might have introduced a selection bias. Fourth, the binary questionnaire used for CKD awareness, while validated in previous large epidemiological studies [17], might have misclassified some participants. The specificity of “YES” responses for low eGFR (< 60 mL/min/1.73 m^2^) or proteinuria (≥ 1 +) was very high at 99.9% in this study, reflecting well the state of CKD. On the other hand, those who answered “NO” may have included some potentially aware. Including such a group of individuals in the control group may have biased the improvement in the eGFR slope toward null. This suggests our findings are robust. Further research is needed to determine whether changing the language of the question would affect the sensitivity for CKD awareness.

## Conclusion

Urine tests and nutritional guidance were associated with the occurrence of CKD awareness in participants with CKD. This study suggests that CKD awareness may slow disease progression. Further research is needed to quantitatively assess CKD awareness based on detailed interviews and explore how these practices relate to CKD awareness levels. Moreover, prospective interventional studies are necessary to determine the extent to which CKD awareness prevents CKD progression.

## Supplementary Information

Supplementary file1 (PDF 364 KB)

## Data Availability

The data underlying this article are available from the corresponding author upon reasonable request.
