# Supplementary material for: Factors associated with awareness of chronic kidney disease, and impact of awareness on renal prognosis
Source: Clin Exp Nephrol. 2024 Dec 16;29(5):596–606. doi: 10.1007/s10157-024-02605-4 (PMC12049403; doi:10.1007/s10157-024-02605-4)
Supplement: Supplementary file 1 — Supplementary file1 (PDF 364 KB) [file 10157_2024_2605_MOESM1_ESM.pdf]

Supplementary Materials

Supplementary Table S1. Medical practice codes for the urine tests and nutritional guidance

	Medical practice code
Urine test	160000310
	160005010
	160159550
Nutritional guidance	113017410
	113017510
	113029810
	113029910
	113030010
	113034710
	113034810
	113034910

Supplementary Table S2. Drug names and drug codes

HT	Drug name	Drug code	Medicinal classification
	Nifedipine	2171014	CCB
	Amlodipine Besilate	2171022	CCB
	Efonidipine	2149034	CCB
	Cilnidipine	2149037	CCB
	Nicardipine Hydrochloride	2149019	CCB
	Nitrendipine	2171020	CCB
	Nilvadipine	2149022	CCB
	Barnidipine	2149030	CCB
	Felodipine	2149035	CCB
	Benidipine	2171021	CCB
	Manidipine	2149027	CCB
	Azelnidipine	2149043	CCB
	Aranidipine	2149038	CCB
	Amlodipine besilate and atorvastatin calcium	2190101	CCB/ HMG-CoA reductase inhibitor
	Amlodipine besilate and atorvastatin calcium	2190102	CCB/ HMG-CoA reductase inhibitor
	Amlodipine besilate and atorvastatin calcium	2190103	CCB/ HMG-CoA reductase inhibitor
	Amlodipine besilate and atorvastatin calcium	2190104	CCB/ HMG-CoA reductase inhibitor
	Telmisartan, amlodipine and hydrochlorothiazide	2149122	CCB/ARB/ Thiazide diuretics
	Losartan potassium and hydrochlorothiazide	2149110	ARB/Thiazide diuretics
	Valsartan and hydrochlorothiazide	2149112	ARB/Thiazide diuretics
	Losartan potassium	2149039	ARB
	Candesartan	2149040	ARB
	Valsartan	2149041	ARB
	Telmisartan	2149042	ARB
	Olmesartan	2149044	ARB
	Irbesartan	2149046	ARB
	Azilsartan	2149048	ARB

Candesartan cilexetil and hydrochlorothiazide	2149111	ARB/Thiazide diuretics
Telmisartan and hydrochlorothiazide	2149113	ARB/Thiazide diuretics
Irbesartan and trichlormethiazide	2149119	ARB/Thiazide diuretics
Valsartan and amlodipine besilate	2149114	ARB/CCB
Olmesartan medoxomil and azelnidipine	2149115	ARB/CCB
Candesartan cilexetil and anmlodipine besylate	2149116	ARB/CCB
Telmisartan and amlodipine besilate	2149117	ARB/CCB
Irbesartan and amlodipine besilate	2149118	ARB/CCB
Valsartan and cilnidipine	2149120	ARB/CCB
Azilsartan and amlodipine besilate	2149121	ARB/CCB
Captopril	2144001	ACEI
Enalapril maleate	2144002	ACEI
Alacepril	2144003	ACEI
Lisinopril	2144006	ACEI
Benazepril	2144007	ACEI
Imidapril hydrochloride	2144008	ACEI
Temocapril hydrochloride	2144009	ACEI
Trandolapril	2144011	ACEI
Perindopril erbumine	2144012	ACEI
Delapril hydrochloride	2144004	ACEI
Trichlormethiazide	2132003	Thiazide diuretics
Benzylhydrochlorothiazide	2132006	Thiazide diuretics
Hydrochlorothiazide	2132004	Thiazide diuretics
Indapamide	2149012	Thiazide-like diuretics
Tripamide	2149007	Thiazide-like diuretics
Mefruside	2135001	Thiazide-like diuretics
Sacubitril valsartan sodium hydrate	2190041	Angiotensin receptor neprilysin inhibitor
Atenolol	2123011	β blocker
Bisoprolol fumarate	2123016	β blocker
Bisoprolol	2149700	β blocker
Betaxolol hydrochloride	2149031	β blocker

	Metoprolol tartrate	2149010	β blocker
	Celiprolol hydrochloride	2149029	β blocker
	Propranolol hydrochloride	2123008	β blocker
	Propranolol hydrochloride	2149014	β blocker
	Nadolol	2123015	β blocker
	Carteolol hydrochloride	2123005	β blocker
	Carteolol hydrochloride	2149025	β blocker
	Pindolol	2123009	β blocker
	Nipradilol	2149021	β blocker
	Carvedilol	2149032	αβ blocker
	Bevantolol hydrochloride	2149036	αβ blocker
	Doxazosin mesilate	2149026	α blocker
	Bunazosin hydrochloride	2149015	α blocker
	Clonidine hydrochloride	2149001	α stimulant
	Guanabenz acetate	2149017	α stimulant
	Methyldopa hydrate	2145001	α stimulant
	Aliskiren fumarate	2149047	Renin inhibitor

DM	Drug name	Drug code	Medicinal classification
	Dapagliflozin propylene glycolate hydrate	3969019	SGLT2-i
	Ipragliflozin L-proline	3969018	SGLT2-i
	Empagliflozin	3969023	SGLT2-i
	Canagliflozin hydrate	3969022	SGLT2-i
	Tofogliflozin hydrate	3969021	SGLT2-i
	Luseogliflozin hydrate	3969020	SGLT2-i
	Buformin hydrochloride	3962001	Biguanide
	Metformin hydrochloride	3962002	Biguanide
	Pioglitazone hydrochloride	3969007	Thiazolidine
	Acarbose	3969003	α-glucosidase inhibitor
	Voglibose	3969004	α-glucosidase inhibitor
	Miglitol	3969009	α-glucosidase inhibitor

Teneligliptin and canagliflozin	3969106	SGLT2-i/DPP4-i
Sitagliptin and ipragliflozin	3969107	SGLT2-i/DPP4-i
Empagliflozin and linagliptin	3969108	SGLT2-i/DPP4-i
Pioglitazone hydrochloride and metformin hydrochloride	3969100	Thiazolidine/Biguanide
Mitiglinide calcium hydrate and voglibose	3969102	Glinide/α-glucosidase inhibitor
Alogliptin benzoate and metformin hydrochloride	3969105	DPP4-i/Biguanide
Vildagliptin and metformin hydrochloride	3969104	DPP4-i/Biguanide
Anagliptin and Metformin hydrochloride	3969109	DPP4-i/Biguanide
Alogliptin benzoate and pioglitazone hydrochloride	3969103	DPP4-i/Thiazolidine
Pioglitazone hydrochloride and glimepiride tablets	3969101	Thiazolidine/Sulfonylurea
Alogliptin benzoate	3969012	DPP4-i
Saxagliptin hydrate	3969017	DPP4-i
Sitagliptin phosphate hydrate	3969010	DPP4-i
Teneligliptin hydrobromide hydrate	3969015	DPP4-i
Linagliptin	3969014	DPP4-i
Anagliptin	3969016	DPP4-i
Vildagliptin	3969011	DPP4-i
Omarigliptin	3969025	DPP4-i
Trelagliptin succinate	3969024	DPP4-i
Nateglinide	3969006	Glinide
Mitiglinide calcium hydrate	3969008	Glinide
Repaglinide	3969013	Glinide
Chlorpropamide	3961004	Sulfonylurea
Acetohexamide	3961001	Sulfonylurea
Glyclopyramide	3961002	Sulfonylurea
Gliclazide	3961007	Sulfonylurea
Glibenclamide	3961003	Sulfonylurea
Glimepiride	3961008	Sulfonylurea
Liraglutide	2499410	GLP-1 receptor agonist
Exenatide	2499411	GLP-1 receptor agonist
Lixisenatide	2499415	GLP-1 receptor agonist

	Dulaglutide	2499416	GLP-1 receptor agonist
	Semaglutide	2499418	GLP-1 receptor agonist
	Semaglutide	2499014	GLP-1 receptor agonist
	Insulin human	2492413	Insulin
	Insulin degludec and insulin aspart	2492500	Insulin
	Insulin degludec	2492419	Insulin
	Insulin detemir	2492417	Insulin
	Insulin glulisine	2492418	Insulin
	Insulin Human	2492403	Insulin
	Insulin glargine	2492416	Insulin
	Insulin aspart	2492415	Insulin
	Insulin lispro	2492414	Insulin
	Insulin glargine and lixisenatide	3969501	Insulin/GLP-1 receptor agonist
	Insulin degludec and liraglutide	3969500	Insulin/GLP-1 receptor agonist

Dyslipidemia	Drug name	Drug code	Medicinal classification
	Atorvastatin calcium	2189015	HMG-CoA reductase inhibitor
	Pitavastatin calcium	2189016	HMG-CoA reductase inhibitor
	Rosuvastatin calcium	2189017	HMG-CoA reductase inhibitor
	Simvastatin	2189011	HMG-CoA reductase inhibitor
	Pravastatin sodium	2189010	HMG-CoA reductase inhibitor
	Fluvastatin sodium	2189012	HMG-CoA reductase inhibitor
	Fenofibrate	2183006	Fibrate
	Bezafibrate	2183005	Fibrate
	Clofibrate	2183002	Fibrate
	Pemafibrate	2183007	Fibrate
	Ezetimibe	2189018	Cholesterol transporter inhibitor
	Omega-3-acid ethyl esters	2189019	n-3 polyunsaturated fatty acids
	Ethyl icosapentate	3399004	n-3 polyunsaturated fatty acids
	Ethyl icosapentate	2189021	n-3 polyunsaturated fatty acids
	Colestimide	2189014	Anion exchange resin

	Colestyramine	2189009	Anion exchange resin
	Probucol	2189008	Probucol
	Niceritrol	2189005	Nicotinic acid derivative
	Tocopherol nicotinate	2190006	Nicotinic acid derivative
	Nicomol	2189004	Nicotinic acid derivative
	Evolocumab	2189401	Proprotein convertase subtilisin/kexin type 9 inhibitor
	Amlodipine besilate and atorvastatin calcium	2190101	CCB/HMG-CoA reductase inhibitor
	Amlodipine besilate and atorvastatin calcium	2190102	CCB/HMG-CoA reductase inhibitor
	Amlodipine besilate and atorvastatin calcium	2190103	CCB/HMG-CoA reductase inhibitor
	Amlodipine besilate and atorvastatin calcium	2190104	CCB/HMG-CoA reductase inhibitor
	Pitavastatin calcium and ezetimibe	2189103	HMG-CoA reductase inhibitor/Cholesterol transporter inhibitor
	Ezetimibe and atorvastatin	2189101	HMG-CoA reductase inhibitor/Cholesterol transporter inhibitor
	Ezetimibe and rosuvastatin	2189102	HMG-CoA reductase inhibitor/Cholesterol transporter inhibitor
Immunosuppressant	Drug name	Drug code	Medicinal classification
	Prednisolone	2456001	Glucocorticoid
	Prednisolone	2456002	Glucocorticoid
	Prednisolone	2456405	Glucocorticoid
	Methylprednisolone	2456003	Glucocorticoid
	Methylprednisolone Sodium Succinate	2456400	Glucocorticoid
	Methylprednisolone Acetate	2456402	Glucocorticoid
	Azathioprine	3999005	Immunosuppressive agent
	Mizoribine	3999002	Immunosuppressive agent
	Mycophenolate Mofetil	3999017	Immunosuppressive agent
	Ciclosporin	3999004	Immunosuppressive agent
	Tacrolimus Hydrate	3999014	Immunosuppressive agent
	Hydroxychloroquine Sulfate	3999038	Immunosuppressive agent
	Rituximab	4291407	Immunosuppressive agent
	Rituximab	4291439	Immunosuppressive agent
	Rituximab	4291451	Immunosuppressive agent
	Cyclophosphamide Hydrate	4211002	Immunosuppressive agent
	Cyclophosphamide Hydrate	4211401	Immunosuppressive agent

HT Hypertension, *CCB* Calcium channel blockers, *HMG-CoA* 3-hydroxy-3-methylglutaryl coenzyme A, *ARB* Angiotensin receptor blocker, *ACEI* Angiotensin converting enzyme inhibitor, *DM* Diabetes mellitus, *SGLT2-i* sodium-glucose cotransporter 2 inhibitor, *DPP4-i* Dipeptidyl peptidase 4 inhibitor, *GLP-1* glucagon-like peptide 1

Supplementary Table S3. International Classification of Diseases-10 code for chronic kidney disease-related disease.

	ICD-10 code
Rapidly progressive nephritic syndrome	N01
Recurrent and persistent hematuria	N028
Chronic nephritic syndrome	N03
Nephrotic syndrome	N04
Unspecified nephritic syndrome	N05
Hereditary nephropathy, not elsewhere classified	N07
Glomerular disorders in diseases classified elsewhere	N08
Chronic tubulo-interstitial nephritis	N11
Tubulo-interstitial nephritis, not specified as acute or chronic	N12
Obstructive and reflux uropathy	N13
Drug- and heavy-metal-induced tubulo-interstitial and tubular conditions	N14
Other renal tubulo-interstitial diseases	N15
Renal tubulo-interstitial disorders in diseases classified elsewhere	N16
Chronic kidney disease	N18
Unspecified kidney failure	N19
Unspecified contracted kidney	N26
Other disorders of kidney and ureter in other diseases classified elsewhere	N298
Postprocedural renal failure	N990
Type 1 diabetes mellitus with renal complications	E102
Type 2 diabetes mellitus with renal complications	E112
Malnutrition-related diabetes mellitus with renal complications	E122
Other specified diabetes mellitus with renal complications	E132
Unspecified diabetes mellitus with renal complications	E142
Disorders of amino-acid transport	E720
Hypertensive renal disease with renal failure	I120
Hypertensive renal disease without renal failure	I129
Hypertensive heart and renal disease with (congestive) heart failure	I130
Hypertensive heart and renal disease with renal failure	I131
Hypertensive heart and renal disease with both (congestive) heart failure and renal failure	I132
Hypertensive heart and renal disease, unspecified	I139
Polycystic kidney, autosomal recessive	Q611
Polycystic kidney, autosomal dominant	Q612
Dependence on renal dialysis[a]	Z992
Kidney transplant status[a]	Z940
Kidney donor[a]	Z524

[a]We assigned Z992, Z940, and Z524 as kidney failure with replacement therapy (KFRT)-related codes.

Supplementary Table S4. Baseline characteristics of participants with CKD

Variables	Total<br>N=13,489	Aware at baseline<br>N=372	Unaware at baseline<br>N=13,117
Male, n (%)	10,097 (74.9)	308 (82.8)	9,789 (74.6)
Age, years (mean ± SD)	53.4 ± 6.8	51.4 ± 8.1	53.4 ± 6.8
Body mass index, kg/m^2 (mean ± SD)	24.2 ± 3.6	23.9 ± 4.1	24.2 ± 3.6
Systolic blood pressure, mmHg (mean ± SD)	124 ± 17	126 ± 19	124 ± 17
Diastolic blood pressure, mmHg (mean ± SD)	78 ± 12	79 ± 11	78 ± 12
Triglycerides, mg/dL (mean ± SD)	125.3 ± 90.0	130.7 ± 84.7	125.1 ± 90.2
HDL-C, mg/dL (mean ± SD)	61.1 ± 17.3	59.1 ± 18.6	61.1 ± 17.3
LDL-C, mg/dL (mean ± SD)	128.1 ± 30.9	111.4 ± 30.3	128.6 ± 30.8
Fasting plasma glucose, mg/dL (mean ± SD)	99.8 ± 17.7	101.6 ± 27.1	99.8 ± 17.4
Serum creatinine, mg/dL (mean ± SD)	1.18 ± 0.94	4.49 ± 4.18	1.08 ± 0.31
eGFR, mL/min/1.73 m^2 (mean ± SD)	53.4 ± 7.73	28.1 ± 20.1	54.2 ± 5.60
eGFR category, mL/min/1.73 m^2, n (%)			
<30	286 (2.1)	186 (50.0)	100 (0.8)
30–39	286 (2.1)	56 (15.1)	230 (1.8)
40–49	1,773 (13.1)	55 (14.8)	1,718 (13.1)
50–59	11,144 (82.6)	75 (20.2)	11,069 (84.4)
Dipstick proteinuria category, n (%)			
Missing data	5,255 (39.0)	140 (37.6)	5,115 (39.0)
Negative (-) or trace (±)	7,554 (56.0)	114 (30.6)	7,440 (56.7)
Positive (≥1+)	680 (5.0)	118 (31.7)	562 (4.3)
Current smoker, n (%)	2,339 (17.3)	71 (19.1)	2,268 (17.3)
Diabetes mellitus, n (%)	1,139 (8.4)	67 (18.0)	1,072 (8.2)
Dyslipidemia, n (%)	7,964 (59.0)	232 (62.4)	7,732 (58.9)
Hypertension, n (%)	5,049 (37.4)	269 (72.3)	4,780 (36.4)
Urine test at least once in a year, n (%)	2,397 (17.8)	76 (20.4)	2,321 (17.7)
Nutritional guidance at least once in a year, n (%)	226 (1.7)	47 (12.6)	179 (1.4)
CKD-related disease, n (%)	1,614 (12.0)	316 (84.9)	1,298 (9.9)

Data are expressed as the frequency (%) or mean ± standard deviation (SD).

HDL-C high-density lipoprotein cholesterol, *LDL-C* low-density lipoprotein cholesterol, *eGFR* estimated glomerular filtration rate, *CKD* chronic kidney disease

Supplementary Table S5. Multivariable logistic regression analysis, stratified by either the urine test or nutritional guidance category

Clinical care	Unadjusted		Adjusted[a]	
	OR (95% CI)	*P*-value	OR (95% CI)	*P*-value
**Urine tests, times/year**				
1–3	2.55 (1.62–4.00)	<0.001	1.96 (1.21–3.17)	0.007
4−6	6.19 (3.24–11.8)	<0.001	2.97 (1.43–6.17)	0.004
≥7	4.53 (2.06–9.97)	<0.001	1.22 (0.48–3.12)	0.671
**Nutritional guidance, times/year**				
1	6.05 (2.18–16.8)	0.001	2.55 (0.79–8.31)	0.119
≥2	19.1 (9.57–38.3)	<0.001	3.38 (1.27–9.04)	0.015

Odds ratios (OR) of the association of the occurrence of awareness with the number of urine tests or nutritional guidance sessions. [a]We adjusted for age, sex, estimated glomerular filtration rate (eGFR), previous CKD-related disease, DM, HT, dyslipidemia, body mass index (BMI), urinary protein, and smoking.

CI confidence interval

Supplementary Table S6. Participants included in the urine tests and nutritional guidance categories

Urine test (times/year)	N	%
0	10,616	82.4
1−3	1,721	13.4
4−6	295	2.3
≥7	254	1.97
**Nutritional guidance (times/year)**		
0	12,718	98.7
1	90	0.70
≥2	78	0.61

The frequency and proportion of participants in each category of the urine tests and nutritional guidance conducted during the year leading up to the next health check-up.

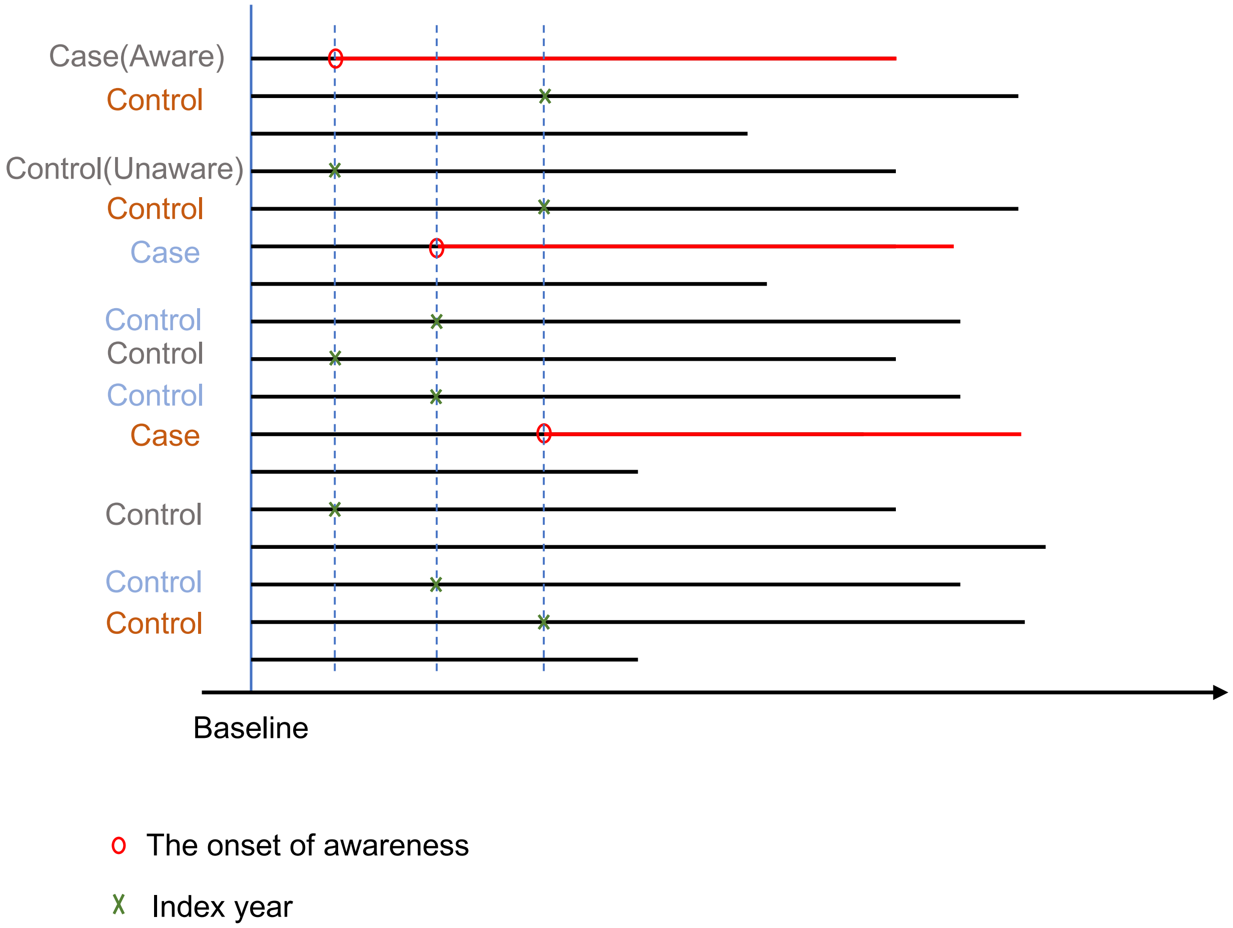

Supplementary Fig. S1 Methods of incidence density sampling.

The cases were participants with the occurrence of awareness. The control group was randomly selected (1:10) from a group of participants who did not develop awareness and were matched to the case group in age (±3 years), sex, eGFR (±5 mL/min/1.73 m^2), urinary protein categories (≥1+, ± / -, or missing), and follow-up period. The index year was defined as the year in which the patient became aware of chronic kidney disease (CKD).

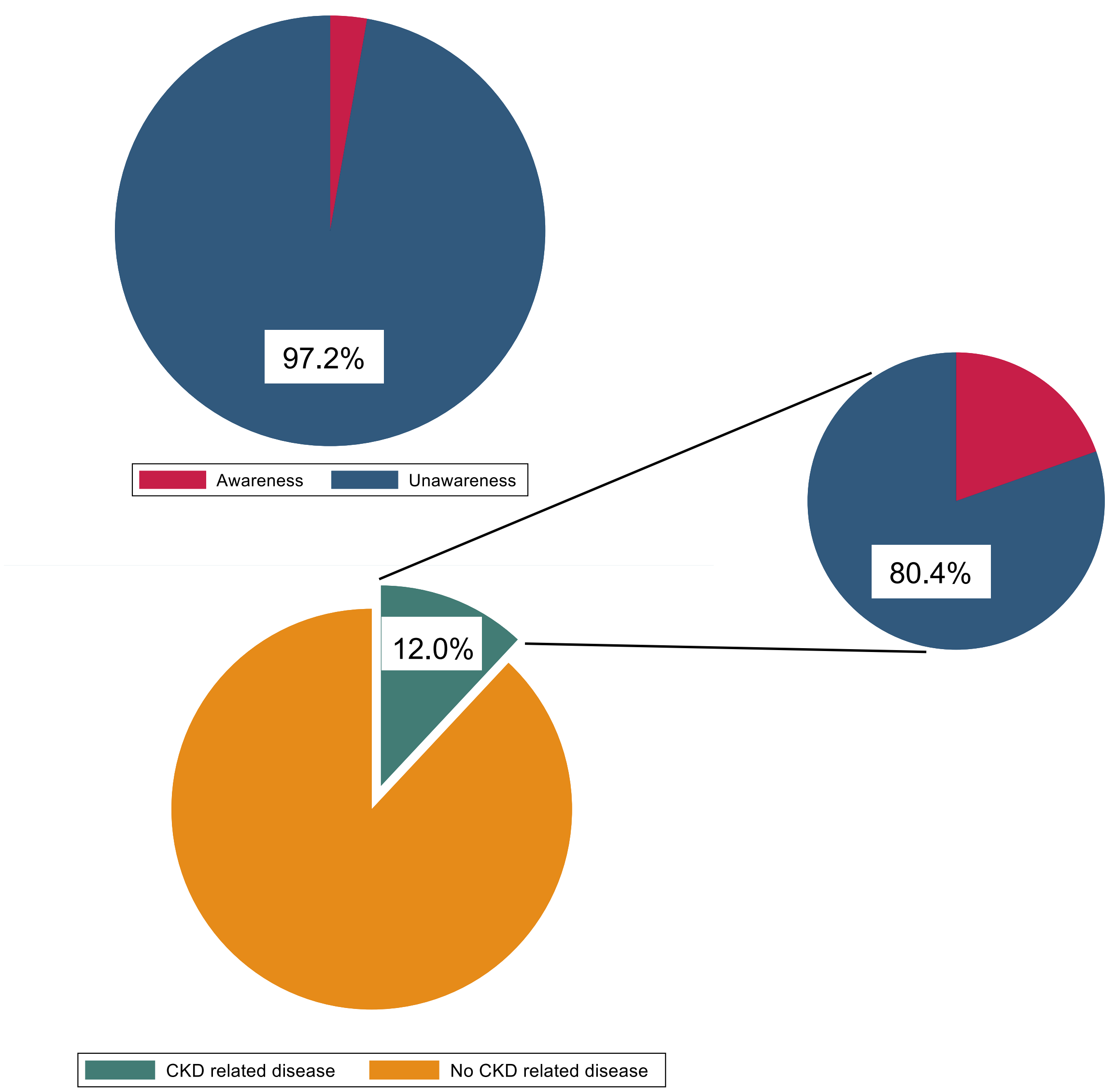

Supplementary Fig. S2 The prevalence of CKD awareness and CKD-related disease codes at baseline.

CKD chronic kidney disease

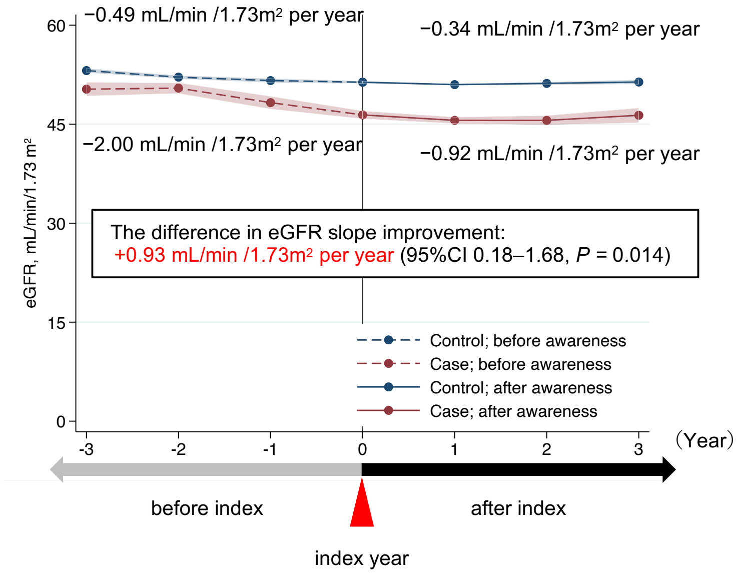

eGFR slope (mL/min /1.73 m² per year) (95%CI)

	Before index	After index	Change in eGFR slope	
Aware (Case)	−2.00 (−3.19 – −0.82)	−0.92 (−2.06 – 0.21)	+ 1.08	+ 0.93
Unaware (Control)	−0.49 (−1.55 – −0.57)	−0.34 (−1.42 – 0.74)	+ 0.15	

Supplementary Fig. S3 Difference in the change of the eGFR slopes between case and control groups before and after the occurrence of awareness in sensitivity analysis.

We adjusted for time-updated age, sex, diabetes mellitus, hypertension, time-updated body mass index, time-updated use of SGLT2-i, RAS-i, glucocorticoids, and immunosuppressive agents, and CKD-related disease codes that were newly added during the year preceding the index year, with interactions between the respective covariates and time. The eGFR in the index year was added without any interaction with time. The blue line represents the unaware (control) group and the red line represents the aware (case) group. The dotted and solid lines show the eGFR slope before and after the index year, respectively. *eGFR* estimated glomerular filtration rate, *CI* confidence interval, *SGLT2-i* sodium-glucose cotransporter 2 inhibitor, *RAS-i* renin-angiotensin system inhibitor, *CKD* chronic kidney disease
